# Sexual Issues in Treating Trauma Survivors

**DOI:** 10.1007/s11930-014-0034-6

**Published:** 2014-12-17

**Authors:** Aline P. Zoldbrod

**Affiliations:** 1 Lexington, MA USA; 2 Affiliate faculty, University of Michigan Sexual Health Certificate Program, Ann Arbor, MI USA

**Keywords:** Developmental sexual trauma (DST), Family of origin, Milestones of Sexual Development, Body Map, Touch, Trust, Overt sexual abuse, Sexual compulsivity, Family violence, Emotional neglect

## Abstract

The effect of interpersonal trauma on sexuality can be profound. The field of sexual trauma is complex empirically and clinically, with contradictory theories and conflicting data. Research definitions and treatment protocols for child sexual abuse are very imprecise. There are no firm, empirically proven guidelines for treating men and women who have been sexually abused as children or adolescents. Overt sexual abuse (OSA) in children and adolescents is defined here as molestation, rape, or incest. Research has shown that OSA may, but does not necessarily, lead to sexual dysfunction in adulthood. The effects of OSA are worsened by concurrent types of family of origin abuse, such as emotional abuse or physical abuse. One factor that seems related to the varying impact of OSA on adult sexuality is the patients’ family of origin experience with nonsexual Milestones of Sexual Development. Without positive experiences with touch, trust and empathy, the ability to relax and be soothed, and power, the effects of OSA are potentiated and complicated. Sexuality is embodied, so experiences with touch are particularly important when working with OSA. A three-color Body Map technique which assesses stored associations to touch is provided. The concept of developmental sexual trauma (DST) is introduced as a way to label traumagenic family events which potentiate OSA or negatively effect sex but which are not explicitly sexual in origin. Strategies to assess and treat OSA are reviewed. Body Maps are recommended to assess and treat sexual trauma.

## Introduction

“It’s really hard to be in my body in the presence of other people and feel safe.”–57-year-old woman, married, presenting for sex therapy, and survivor of emotional, physical, and sexual abuse.

Sexual trauma, in all its forms, is common in the USA and, indeed the world [1], with negative implications for the optimal sexual health, pleasure, and functioning of male and female survivors. Being able to have intimate, engaged sexual encounters is critical to having vital romantic relationships [2, 3]. The primary focus of this paper is overt sexual abuse (OSA): child and adolescent molestation, rape, and incest. However, assessing and treating sexual trauma is complicated. Researchers and clinicians consistently have pointed out the negative effect of all forms of childhood maltreatment on adult sexual functioning [4–6]. As research continues, it is clear that other kinds of abuse in the family, including neglect, physical abuse, emotional abuse, and witnessing physical violence, are quite common [7•]. They potentiate the psychological and sexual effects of OSA. These other kinds of abuse can be considered developmental sexual trauma (DST). They damage adult sexuality by affecting the patients’ feelings, associations, and implicit memories about touch, trust, safety, power, and gender.

## Overt Sexual Abuse

Overt sexual abuse (OSA) is the intentional arranged participation of the child in sexual activities which are developmentally inappropriate and for which the child cannot give informed consent and, in adolescents, rape.

Accurate prevalence statistics come from large studies collected by California HMOs. Dube et al. [8] published a 2005 study of 17,337 adult HMO members using a control group as well as controlling for exposure to other kind of childhood trauma. They found contact child sexual abuse reported by 16 % of males and 25 % of females. OSA statistics, research, and thus clinical understanding are hampered by differing definitions of sexual contact, huge variations in the intensity, type, and longevity of the abuse, vast differences in the family environments the children come from, inconsistent definitions of the end of childhood, lack of control groups, and questions about the veracity of retrospective recall by adults [9, 10]. Probably because of these definitional problems, one meta-analysis concluded that the harm caused by child sexual abuse was not necessarily intense or pervasive [11].

Incest is OSA perpetrated by a family member. Children’s symptoms are more severe when sexual abuse is by parent figures [12], when it involves penetrative sex, when it is accompanied by aggression, when there are several abusers, and/or when it continues over months or years [5, 13, 14]. Finkelhor [15] writes brilliantly about the betrayal the child experiences and about the child’s sexualization, powerlessness, stigmatization, and change in worldview. Maternal support has been shown to be the most crucial factor in children’s recovery from child sexual abuse [16, 17].

In adults, OSA has been correlated with higher levels of depression, guilt, shame, and self blame, all of which are likely to affect comfort with sexuality. In a carefully constructed study of adult survivors of OSA, male and female OSA survivors had significant social and relationship problems and suicidality [8], and women had lowered self-esteem and decreased life satisfaction [18].

Maltz [19] described the most common sexual symptoms stemming from sexual abuse as follows:

Avoiding, fearing, or lacking interest in sex; approaching sex as an obligation; experiencing negative feelings such as anger, disgust, or guilt with touch; having difficulty becoming aroused or feeling sensation; feeling emotionally distant or not present during sex; experiencing intrusive or disturbing sexual thoughts and images; engaging in compulsive or inappropriate sexual behaviors; vaginal pain or orgasmic difficulties in women; and erectile, ejaculatory, or orgasmic difficulties in men.

Sexual abuse research has been focused primarily on females, children, and adults. Less attention has been focused on adolescents and men. Some excellent research exists on the sexual sequellae of female survivors of OSA and corroborates Maltz. However, it should be noted that researchers examining the association of OSA with women’s sexual functioning describe conflicting results, with some studies finding very modest effects [20]. Several studies have found that OSA histories in women are more associated with problems with sexual satisfaction rather than sexual function per se [9, 10, 21].

In studies, OSA survivors were found to have distortions in sexuality [22, 23] including heightened sexual activity and permissive attitudes [24, 25] (e.g., excessive masturbation and an overactive sexual fantasy life); sexual risk-taking behaviors [26]; early pregnancy [27]; compulsive sexual behaviors [28, 29], e.g., prostitution [30]; sexual avoidance [31]; anxiety and fear of being sexual [32]; changed self schemas and negative affect during sexual arousal [33, 34]; and sexual dysfunction [9, 35]. Finally, women OSA survivors frequently present with somatic chronic pelvic pain [36–38].

There are far fewer studies of the sexual sequellae of OSA in men than in women. The research which exists documents a significant negative effect on sexuality, with males more likely to act out sexually and to engage in other self-destructive behavior [20]. Significant correlations between OSA and sexual dysfunction are found [39, 40]. In a national probability sample of 1749 women and 1410 men aged 18 to 59 years, Laumann, Paik, and Rosen [34] found that for male survivors, OSA had significant sexual effects. For men who were touched sexually before puberty, all categories of sexual dysfunction were more common than in men who had not been sexually abused. Forceful penetrative sex is associated with high-risk sexual behaviors [41]. One large study of 1002 male college students found that male victims are much more likely to experience adult sexual assault than nonvictims. [42]. Male socialization and shame prevent men from acknowledging OSA, hindering healing [43•]. It appears that some adolescent males eroticize OSA experiences and deny them as abusive. Experiencing OSA may be one factor leading to sexual compulsivity in men [44–46].

### The Importance of the Family in which the Sexual Trauma Occurs: OSA, the Milestones of Sexual Development and Developmental Sexual Trauma

The family environment in which the OSA occurs is a critical determinant of how well survivors fare [7•, 47]. Powerful, recent research indicates that most survivors of serious OSA come from dysfunctional families where usually there are other adverse childhood experiences—physical abuse, neglect, emotional abuse, family violence, or alcohol or drug abuse [48]. Research consistently finds that survivors of OSA fare worse with each type of adverse childhood experience [48–50].

Nonsexual family of origin abuse, such as emotional abuse, causes negative sexual sequellae on its own, separate from OSA [21]. On the other hand, the presence of powerful, positive Milestones of Sexual Development, such as good family experiences with touch, trust, empathy, safety, relaxation, and power, can offset the trauma of some experiences with OSA. The absence of these good family experiences is DST, which intensifies the trauma of OSA.

Good Milestone experiences “link feelings of being loved and feeling ‘good enough’ with other developmentally and sexually crucial abilities and associations, for instance, with:(1) embodied feelings of pleasure, including (appropriate) familiarity with the sights, touches, tastes, and smells of bodily intimacy; (2) the ability to tolerate feelings, one’s own and others’; (3) emotional closeness to another; (4) relaxation, trust, safety, and energy flow; (5) the expression of feelings; and (6) ultimately, with the free expression of sexuality [51]” and sexual satisfaction.

## Good and Bad Associations to Touch, OSA, and Trauma

Good associations to touch are a crucial Milestone of Sexual Development that is subverted by OSA. Untraumatized children crave touch, sensation, and close physical intimacy [52]. Researchers [53 p 124] spoke with 31 children aged 8 to 9 years, along with their parents about cuddling and being in love. “Almost all of them regarded cuddling as something positive, either because of the bodily sensations or because of the feeling of safety it gives them (p 123).” The childrens’ associations to cuddling were safe, nice, soft, cheerful, fun, kind, and comforting. They reported having many areas in their body in which touch felt pleasant and several areas (none of them erogenous) where touch felt exciting. More than half of them deemed themselves to have been in love. Their associations to being in love were positive and made reference to pleasant physical sensations.

When associations to touch are damaged in childhood, patients’ ability to experience sexual pleasure is seriously impacted. In a study of 1000 French men and women, touch was the most important of all the senses for sexual pleasure for both men and women, and foreplay was “important, very important, or essential” for 89.1 % of subjects [54]. Research and clinical literature indicates, at least for non-abused women, that receiving pleasurable touch that does not lead to intercourse and sufficient pleasurable touch prior to intercourse leads to good sexual function [55, 56]. Good associations to touch are subverted by OSA, DST, and family violence. Traumatic experiences with touch are stored in the body as implicit memories and unpleasant sensations, split off from conscious awareness [57••, 58, 59•]. Experiencing family physical violence toward oneself or watching violence between parents is common among families where there is OSA, potentiates OSA, and has its own sexual sequellae.

## Body Maps in Evaluating and Treating Trauma and Stored Negative Sensation

**Fig. 1:**
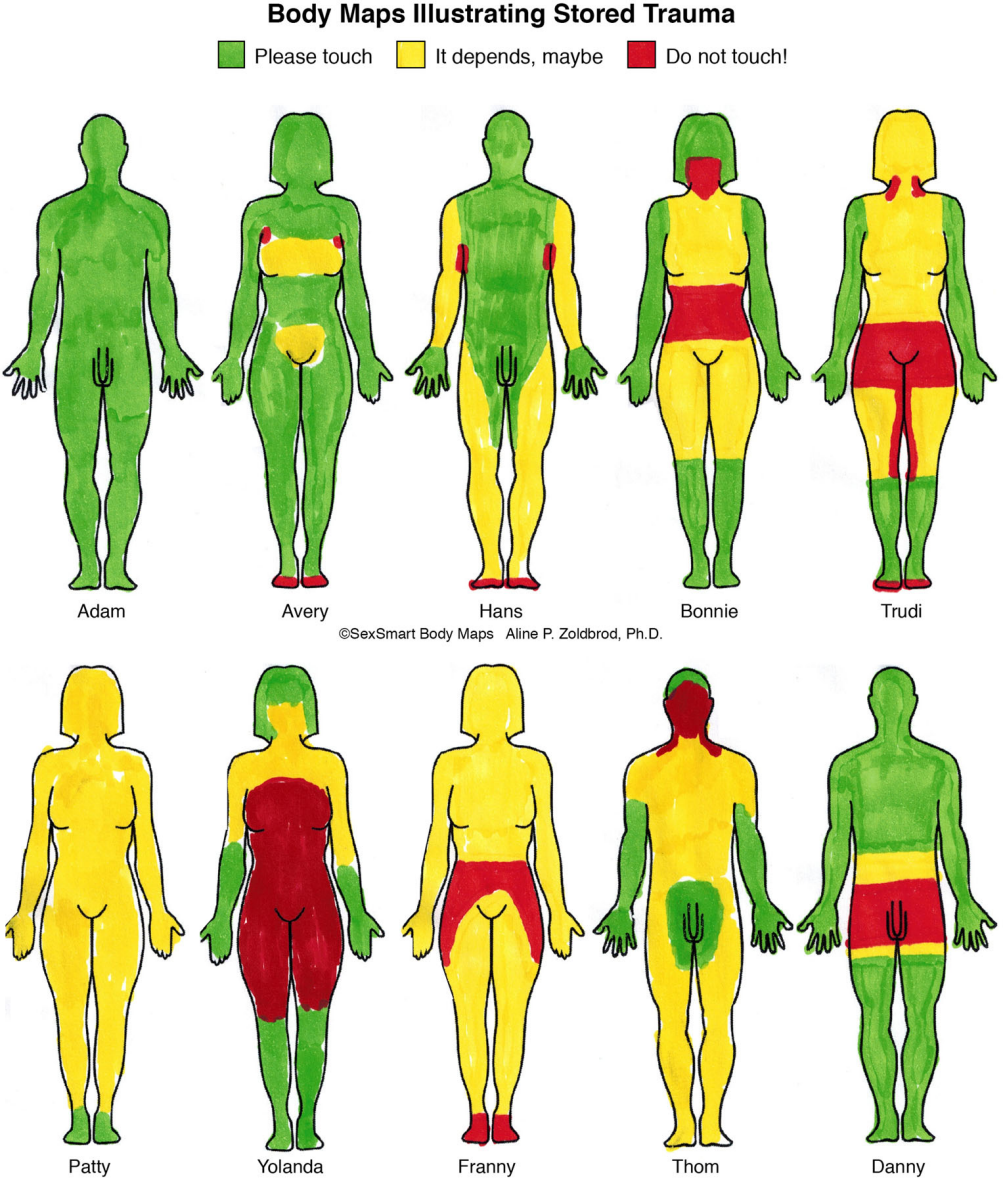
Body Maps

In evaluating and treating sexual trauma, it is useful to have patients draw three-color Body Maps, which assess stored implicit and conscious trauma [4] (see Fig. 1 and Table 1). I devised this clinical tool in the 1990’s. Patients are asked to draw an outline of the front and the back of their body, and thinking of being touched by someone they love, to color in the map. The color code is the same as a traffic light: green means go; yellow means caution or it depends; red means no. Most survivors of OSA will not be surprised to find their genitals, buttocks, anus, or their mouths red, but they may well be surprised to see how the bad feelings have spread to the rest of their body (often indicating concurrent DST). Some OSA patients have dissociated the trauma memories, but the Body Maps alert the professional to the occurrence of trauma. Survivors of DST may well be bewildered by what their Body Maps evidence, since they do not count what happened to them as abuse. Memories of gender microaggressions [60] from strangers and peers also show up on Body Maps.

**Table 1:** How To Use Body Map to Screen for Hidden and Stored Trauma ©Aline P. Zoldbrod

Color key: green = please touch; yellow = it depends; red = no!			
Possible trauma condition	Typical color scheme	Issues to treat explore/ask	Example (see figure by name)
Typical man with no trauma	Almost entirely green, perhaps small areas of red and yellow	• Explore any yellow or red areas; may indicate injury, illness, uncomfortable feelings about body parts.	Adam
Typical woman with no trauma	Almost all green. Breasts, pudendum, and anus typically yellow Note: women often color primary erogenous zones yellow because they prefer to become aroused with whole body touches before having primary erogenous zones touched. Small amounts of red and yellow may be present.	• Explore as above. • Be sure to decode any red or yellow areas—may indicate having been touched in an unpleasant way, pain or injury, or body image issues.	Avery
No trauma. But family sex-negative. Family did not touch.	Green and some yellow. Perhaps small red areas	• Hans actually has become much more comfortable with touch as an adult. In his drawing of himself as an adolescent, his genitals were yellow. His wife taught him about the pleasures of being touched. His red areas are where he is ticklish.	Hans
Physical, emotional and verbal abuse (DST) No OSA	Often not much green. Lots of yellow. Red often (but not always) in areas of physical abuse. Much smaller green areas relative to red and yellow areas than in patients with no trauma.	• Where you see large areas of red, or large areas of yellow, be mindful, cautious. • Do not open up trauma memories before you have worked to give patient inner resources to handle upsetting sensations. • It is not unusual to see red on womens’ abdomens without physical abuse there. May mark dislike of belly fat. • Bonnie was treated very harshly and her mother scratched her face on several occasions. • Patty was hit occasionally, and received constant emotional and verbal abuse. • Frannie had physical and emotional abuse and physical neglect. • Thom was constantly emotionally abused and hit on face and shoulders.	Bonnie Franny Patty Thom
Idiosyncratic Body Map: emotional neglect, physical and emotional abuse (No OSA)	The red on his genitals is unusual for a man with no OSA history.	• Danny came from a home with alcoholism and physical violence, but his mother was loving and affectionate. He feels insecure about his weight and his penis size. He has low desire and avoids sex.	Danny
Witness of family violence toward other family members (No OSA)	Mixed green, yellow, and red	• The patient bears witness to violence done to the other family member, often by holding trauma in the same body area where other person was assaulted. • Trudi’s mother was beaten by father repeatedly throughout Trudi’s childhood. Mother stayed in relationship far too long. • Note that genitals are red even without OSA. Trudi has profound ambivalence about being a sexual woman in relationship with a man. She does not want to identify with the gender of the victim of violence, her mother.	Trudi
OSA and DST	Red on genitals from OSA.	• Yolanda had intrafamilial sexual abuse that continued for a long time, as well as physical and emotional abuse.	Yolanda
Other possible explanation: Gender microaggression outside of family	Mixed color scheme not accounted for by any intrafamilial trauma	• Person receives demeaning sexual, gender or body-focused comments. • Preadolescent girls whose breasts develop early and adolescent girls with large breasts are targets of hostile, sexual, objectifying comments. • Boys targeted because of height or penis size. • GBLT adolescents are targets of shaming, hostile comments about height, gait, gender-variant appearance. • For all, resulting feelings of shame and confusion are stored in body.	No example provided in Body Map graphic

## Treating OSA

Because different instances of OSA vary so much, there is no specific “sexual abuse syndrome” among survivors, nor has any specific treatment model has been proven efficacious [61••]. When a patient reveals a history of sexual abuse, the clinician should not presume to know what its effects are.

It is critical to screen for all kinds of family of origin traumatic experiences, including DST. Use of a screening tool such as the Childhood Trauma Questionnaire [62] is recommended. Most screening tools do not ask about exposure to pornography, and including this variable is worthwhile.

The Body Map exercise described herein indicates whether the patient has any nonverbal, implicit, feared sensations or memories of trauma. If there is very little green on the Body Map, the therapist must proceed cautiously. The data evidenced on the Body Map may not be consciously known to the patient. Before processing the Body Map, it is critical that a therapeutic alliance be established and that patients have developed the internal resources to tolerate the sensations and the memories of the trauma [63–65]. The clinician should assess for suicidality and dissociative phenomena. If the patient clearly has complex trauma, a dissociative disorder, or is at risk for suicide, a referral out may be appropriate.

Newer research reviewed by Rellini [61••] shows that interventions that use mindfulness contribute to healing OSA [66]. This is congruent with current trauma research, which finds that mindfulness aids emotional regulation and can lead to changes in brain regions related to body awareness and fear [57••].

Maltz [19, 67, 68] suggests that a first step in healing is to help the survivor to connect their current sexual problems with their past sexual trauma. Maltz stresses the importance of the survivor gaining skills in assertiveness and self awareness. If the survivor is in a committed relationship, their partner can be an active participant in the healing process. Establishing choice, trust, respect, safety, and equality in the relationship is an important precondition to couples work going forward. Other OSA literature remains relevant and useful [69–71], employing case studies, carefully staged treatment and an emphasis on safety, and maintaining the survivor’s sense of agency and power in the present.

Because childhood sexual abuse has been correlated with higher levels of depression, guilt, shame, and self blame, all of which are likely to affect comfort with sexuality, researchers have looked at altering self schemas of survivors of OSA [35]. There is a huge role for psychoeducation, support groups, [72••], and group therapy in addressing self blame and shame. When the sexual abuse was inflicted by an esteemed trusted adult, it may have been hard for the survivor to see the perpetrator through adult eyes, making it difficult for them not to blame themselves. In a group, each participant can see that the other group member’s perpetrator was at fault, identify with the other person, and let go of their guilt, self blame and shame.

## Some Additional Thoughts on Treating OSA

There is an important piece that is missing from the discussion on treating OSA. Sex is embodied. Enjoying sexuality is not a cognitive process. For some OSA patients, the ones who have very little green on their Body Maps, the body holds the terror [57••, 58]. From this perspective, avoiding sex, fearing arousal, and lack of desire make sense. If there was DST or other types of family trauma, with no positive associations to touch or trust, there may be layer upon layer of stored fear and multiple places in the body where sensation feels dangerous. This becomes evident through patients’ three-color Body Maps.

Where untraumatized children like the ones described in Rademakers’ research [53] can grow into adults who enjoy sex, hugging, and kissing, the OSA survivors we treat feel quite the opposite. Their associations, when asked to make a list of associations to touch, are [4]: “fear,” “controlling,” “out of control,” “pain,” “awkward,” “numb,” “tense/anxiety,” “guilt,” “startle response,” “bad memories,” “discomfort,” “weird,” or “danger.”

If survivors of mild OSA with DST were neglected and never touched lovingly, feelings of vulnerability and the longing to be touched and loved may be too powerful to tolerate (as is their partner’s longing to be cared for). These patients’ associations to touch can be “what does this mean?, “is this proper?,” “numb,” or that “touch is “boring” and a “waste of time.”

Patients with Body Maps without much green avoid sex because the fear of the sensations has been wired into their brains. They are phobic of their own sensations. Being sexual with a partner, not knowing where you might be touched or how you might be touched, is a vulnerable experience. Healing the patient’s fear of being touched, of feeling out of control, of unleashing frightening sensations is often a critical task, and traditional cognitive therapy or sensate focus is unlikely to be therapeutic. In individual, trauma-informed therapy, the therapist can slow down her volume and rate of speech. The therapy focuses on mindfulness, attentiveness to the present, being in one’s body, the expression of emotion, and the therapeutic use of language [59•]. The goal is to get the patient to be curious about what she is feeling, to not avoid emotions and sensations. The environment the therapist creates is one that causes mild to moderate stress. The goal is to expose the patient to some of the upsetting and forbidden emotions and sensations but in a safe and contained way, with the therapist at the patient’s side, helping to construct a narrative about what went on, what the emotions were at the time, and the fact that the patient is now safe.

Some schools of therapy talk about reexperiencing trauma in dual consciousness. The emotions are felt in the body in the present, and they feel frightening and difficult to tolerate. But at the same time, the patient knows that it is old feelings that she is feeling right now. Though the emotions seem vivid and feel intolerable, they do not represent a current danger. So she learns the skill of tolerating and noticing what she feels without automatically acting on the fear. These ideas about how to reprocess trauma involve the ability to reconceptualize a memory based on developing maturity. It is a breakthrough when a female OSA survivor can relax, call up a feared memory, focus, feel the sensations, picture herself as the girl with the terrifying emotions in her body, and speak to her young self as her adult self compassionately and soothingly.

Under the intense terror of severe OSA, the part of the brain that processes in words shuts down [57••, 73]. Through trauma-informed therapy, finally, it is possible to create a new narrative that places the negative emotions and memories attached to the sensations in the past. Over time, the Body Maps are redrawn. As the sensations, emotions, and thoughts get integrated, the Body Maps show more green. At that point, couple sex therapy could be most helpful.

## Summary

Sexual abuse of children and adolescents is a serious problem, with long-lasting consequences for some survivors. Each patient who experienced OSA must be evaluated as a case of one. Because of the huge variations in the definitions and types of OSAs, and differences in the kinds of families patients came from, it is possible that some patients had no ill effects from the trauma.

Evaluation for OSA is complicated and should include taking a complete trauma history, looking at the specifics and the severity of the sexual abuse that occurred, whether the patient had some concurrent good experiences in the Milestones of Sexual Development or had DST, whether or not the trauma was revealed to parents, and how the parents or guardians responded to the revelation. It is critically important to determine whether or not other adverse childhood experiences like experiencing or witnessing physical violence or suffering emotional abuse or neglect occurred in the family of origin, since we know that these events complicate and potentiate the damage of the OSA.

Research indicates that both male and female survivors of OSA have sexual problems stemming from the abuse. Compared to the research on females and children, the research on the effect of OSA on male survivors is relatively sparce. Male socialization and reluctance to label OSA as abuse is evident in the fact that male survivors are less likely to seek help from professionals and to act out instead. Mental health and medical professionals should not assume that male survivors will spontaneously reveal a history of OSA without being queried. Providers should take care not to minimize revelations of OSA by male survivors, since they may already feel stigmatized, confused, and ashamed at being victims.

Some patients with OSA had many other traumatic experiences and may present with complex trauma. Care should be taken not to elicit too much traumatic material until the therapeutic alliance is strong, and patients have the internal resources and the skills to regulate their affect and tolerate and process disturbing feelings, memories, and sensations in dual consciousness.

Research has not proven any specific treatments as effective in the treatment of OSA in either men or women; however, mindfulness techniques do show promise. The current clinical literature does a good job of outlining the issues and describing a thoughtful way to proceed with treatment. The Body Map exercise may be a helpful addition to evaluate the depth of the trauma, and in appropriate patients, can be useful to gauge progress throughout treatment.
